# RAPID perfusion CT in acute ischemic stroke: an educational pictorial review of common patterns, pearls, and pitfalls

**DOI:** 10.1186/s13244-026-02427-w

**Published:** 2026-10-13

**Authors:** Andrea Buoso, Caterina Bernetti, Michele Tondo, Ilenia Barbalace, Teresa Giannarelli, Michele Pillitteri, Andrea Romano, Alessandro Bozzao, Bruno Beomonte Zobel, Carlo A. Mallio

**Affiliations:** 1 Fondazione Policlinico Universitario Campus Bio-Medico, Rome, Italy; 2 Research Unit of Radiology, Department of Medicine and Surgery, Università Campus Bio-Medico di Roma, Rome, Italy; 3 Department of Neurosciences, Mental Health and Sensory Organs, School of Medicine and Psychology, “Sapienza” University, S.Andrea Hospital, Rome, Italy

**Keywords:** Acute ischemic stroke, RAPID, Mismatch, Infarct core, Penumbra.

## Abstract

**Abstract:**

Rapid processing of Perfusion and Diffusion (RAPID) software for CT perfusion (CTP) is a valuable tool for the evaluation of acute ischemic stroke and therapeutic decision-making in extended therapeutic windows. However, automated maps should not be interpreted in isolation. A structured, pattern-based approach, integrating qualitative visual assessment with automated quantitative output and clinical information to refine therapeutic selection, remains essential to mitigate pitfalls and optimize patient care. The aim of this article is to provide an educational pictorial review based on selected illustrative cases of the most common imaging patterns, interpretive pitfalls, and technical features of CTP processed with RAPID software in acute ischemic stroke, that may serve as a practical guide for radiologists and neuroradiologists.

**Key Points:**

***Question*** How can radiologists effectively interpret automated RAPID CT perfusion maps in acute ischemic stroke while avoiding common technical traps, diagnostic pitfalls, and anatomical limitations?

***Findings*** Integrating qualitative visual assessment, quantitative RAPID outputs, and clinical-vascular data within a structured workflow minimizes diagnostic errors and optimizes patient selection for reperfusion.

***Critical relevance statement*** This educational pictorial review provides a practical, pattern-based approach to RAPID CT perfusion interpretation, highlighting clinical applications, technical limitations, software variability, and diagnostic pitfalls to advance everyday neuroradiological practice and personalized stroke management.

**Graphical Abstract:**

**Figure:**
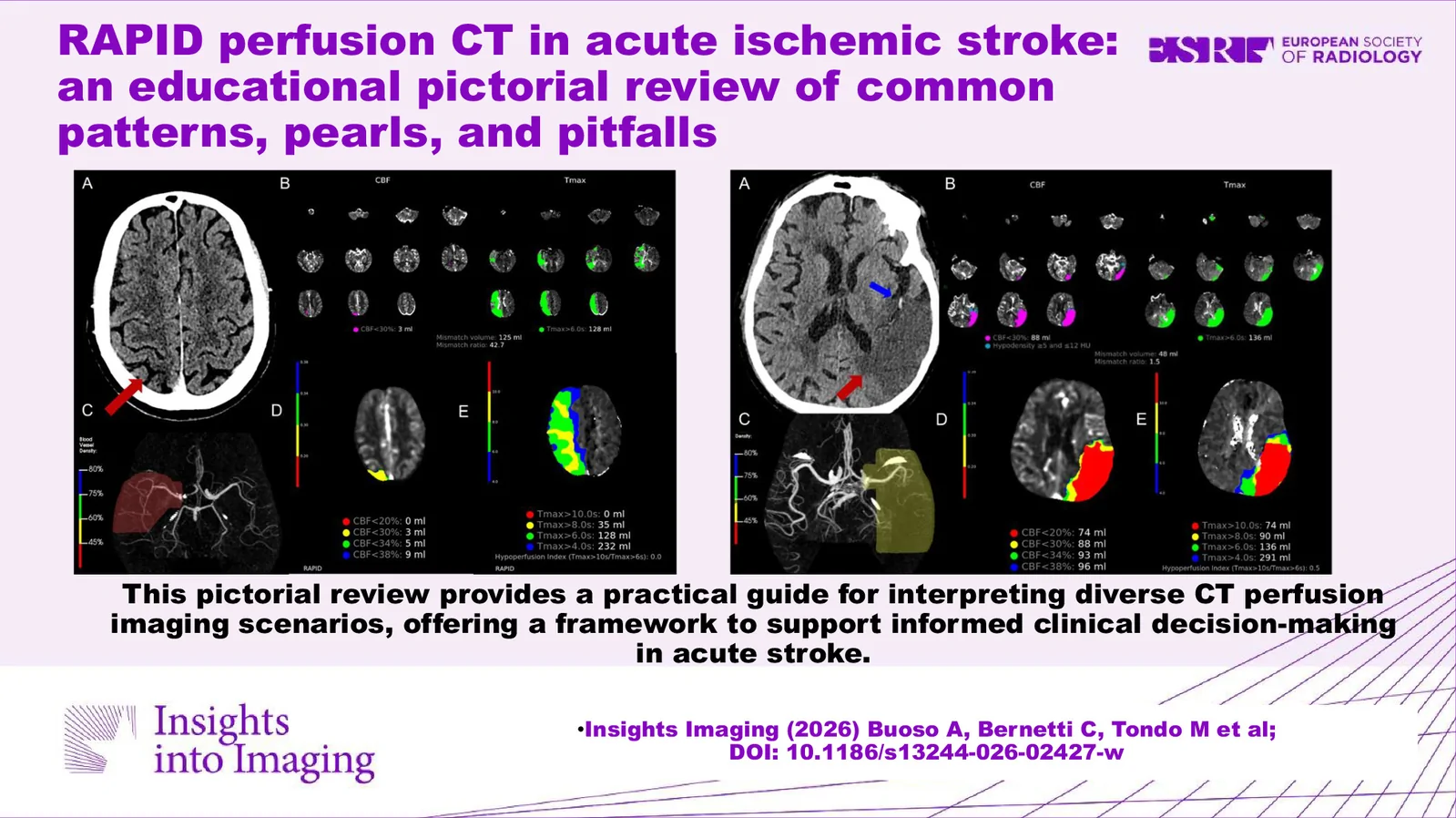

## Introduction

Acute ischemic stroke remains one of the leading causes of death and long-term disability throughout the world. During the hyperacute phase, rapid identification of irreversibly infarcted and hypoperfused but potentially salvageable tissue is critical for selection of patients who may benefit from reperfusion therapy. In the past decade, imaging has evolved from a purely time-based strategy to a “tissue-clock” paradigm, where treatment decisions are driven by advanced imaging hemodynamic biomarkers, rather than time of symptom onset [1].

CT perfusion (CTP) represents a central part of this approach, through quantification of cerebral hemodynamics, it allows discrimination between infarct core and penumbra and, thus, provides a more accurate estimate of tissue viability than non-contrast CT (NCCT) alone [2, 3].

This ability is especially important for patients presenting outside conventional treatment time windows, wake-up strokes, and complex clinical conditions in which initial CT findings are equivocal or non-specific [4, 5]. Implementation of automated post-processing systems such as RAPID has contributed substantially to the general application of perfusion imaging to the acute stroke work-up. With standardized quantitative maps and summary results based on predefined thresholds, including infarct core derived from cerebral blood flow (CBF) and volumes and ratios of mismatch, RAPID provides rapid and reproducible results [6, 7].

These results were critical for major randomized controlled trials in which the benefit of treatment selection based on perfusion imaging was demonstrated. In particular, the clinical importance of selection for mechanical thrombectomy based on mismatch and for extended time windows was established by the DEFUSE 3 and DAWN trials, whereas the application of perfusion-directed thrombolysis for selected patients outside conventional time limits was supported by the EXTEND trial [4, 5, 8].

Despite these advances, perfusion imaging is not without limitations. Automated map interpretation depends on acquisition quality, threshold selection, patient motion, characteristics of contrast bolus, and physiological variability. In addition, the estimated infarct core or penumbra may differ from MRI results or may not fully reflect the final infarct, especially in cases of early reperfusion, collateral variability, or technical artifacts [7, 9, 10]. For these reasons, although automated software improves standardization of workflow, interpretation of images still requires radiological expertise and integration with clinical and vascular imaging results.

The objective of this article is to provide an educational pictorial review based on selected illustrative cases of the most common imaging patterns, interpretative pitfalls, and technical features of CTP with RAPID software in acute ischemic stroke. With correlation to NCCT, perfusion maps, quantitative RAPID results, and, when available, MRI, the goal of this review is to offer a practical approach for identifying typical and atypical perfusion situations appropriate for daily clinical practice.

## Educational case selection

The imaging examples presented in this pictorial review were selected from the institutional imaging archive to illustrate representative CT perfusion patterns, common interpretative pitfalls, and technical limitations encountered during RAPID software analysis in patients with suspected acute ischemic stroke. Cases were chosen exclusively for their educational value and their ability to demonstrate specific imaging concepts relevant to daily neuroradiological practice.

The purpose of this review is to offer a practical and educational visual guide to the interpretation of RAPID-derived perfusion maps. The selected examples highlight clinically relevant scenarios frequently encountered in acute stroke imaging, including classical target mismatch profiles, large mismatch with minimal infarct core, extensive infarct core patterns, intermediate mismatch configurations, threshold-dependent variations in *T*_max_ interpretation, motion-related artifacts, discrepancies between CT perfusion and MRI findings, anatomical limitations in deep gray matter and posterior circulation territories, and common stroke mimickers.

## Principles of RAPID CT perfusion interpretation

Interpretation of CT perfusion studies with RAPID software should not be limited to automated summary volumes and must always be combined with NCCT, CT angiography, clinical findings, and evaluation of image quality [6, 7]. Although RAPID provides standardized quantitative results, accurate interpretation requires appreciation of the physiological basis and limitations of each perfusion parameter [6, 7].

## Infarct core

Estimates of infarct core volume were obtained for regions of cerebral blood flow less than 30% of that measured in the corresponding contralateral hemisphere. This criterion has been extensively validated as a surrogate measure of irreversible ischemic injury and is widely used in major clinical trials of image-directed methods of reperfusion [2, 4, 5]. However, automated infarct core estimates should not be considered as absolute representations of biological reality, because they may be influenced by variations in acquisition protocols, patterns of collateral circulation, degrees of reperfusion, and technical artifacts [7]. As a result, automated infarct core estimates may under- or overestimate the final infarct extent on follow-up imaging [7, 11].

## Hypoperfused tissue and *T*_max_

The degree of hypoperfusion was primarily quantified with maps of time to maximum cerebral blood flow. Application of a threshold of greater than 6 s provided the most widely accepted indication of critically hypoperfused tissue at risk for infarction and formed the basis for most contemporary approaches to treatment based on patterns of mismatch [3, 6]. Use of lower thresholds included regions of benign oligemia, whereas progressively higher limits reflected increasingly severe impairments of cerebral perfusion. Thus, interpretation of maps of time to maximum cerebral blood flow should emphasize overall patterns and severity of delays in cerebral perfusion rather than depend solely on a single value for the duration of hypoperfusion [3, 7].

## Core–penumbra mismatch and collateral circulation

The mismatch concept reflects the contrast between irreversibly damaged tissue and hypoperfused but potentially salvageable brain parenchyma. Patients with relatively small infarct cores and large volumes of hypoperfused tissue exhibit favorable patterns of mismatch and are most likely to benefit from reperfusion therapy [4, 5, 12], especially in extended intervals of treatment. The importance of selecting patients based on patterns of tissue viability was confirmed by major studies in which treatment decisions based on estimates of tissue viability produced greater clinical benefit than conventional criteria of time of treatment [4, 5, 12, 13].

Collateral perfusion is a major determinant of the rate of progression of infarction and of patterns of tissue viability after arterial occlusion. Robust collateral pathways maintain brain parenchymal viability despite severe perfusion delays, whereas poor collateralization accelerates core expansion and leads to worse clinical outcomes [14, 15]. As a result, interpretation of patterns of mismatch must always consider the magnitude and severity of abnormalities of *T*_max_, combined with findings of vascular imaging and the overall clinical situation. The hypoperfusion intensity ratio (HIR) represents an indirect index of collateral efficiency and the severity of hypoperfusion. Lower ratios reflect better collateral status, slower rates of infarct progression, and improved clinical outcome, whereas higher ratios indicate a greater burden of critically hypoperfused tissue and a higher risk of infarct expansion [16]. Therefore, this parameter should augment traditional indices of mismatch and provide additional information concerning the vulnerability of tissue to hypoperfusion and the adequacy of collateral perfusion.

## Practical interpretation workflow

A complete strategy for rapid interpretation should include: (1) assessment of established signs of cerebral ischemia on NCCT; (2) identification of arterial occlusion on CTA; (3) quantitation of volume of infarct core on maps of cerebral blood flow; (4) determination of the volume of hypoperfused brain on maps of time to maximum; (5) evaluation of patterns of mismatch and of features of collateral perfusion; and (6) confirmation of adequacy of image quality and absence of potential artifacts of technical origin [6, 7, 13].

Automated results must always be interpreted in the context of the overall clinical and imaging findings and never considered in isolation [7, 10, 12]. Special attention should be given to artifacts of motion, abnormalities of the contrast bolus, limitations of anatomic coverage (e.g., for the posterior circulation and deep gray matter structures) and potential discordance between findings of perfusion imaging and subsequent MRI results [7, 10, 12]. As a result, RAPID represents a tool for assisting, rather than replacing, the expertise of the radiologist and of the multidisciplinary clinical team [6, 7, 15].

**Fig. 1:**
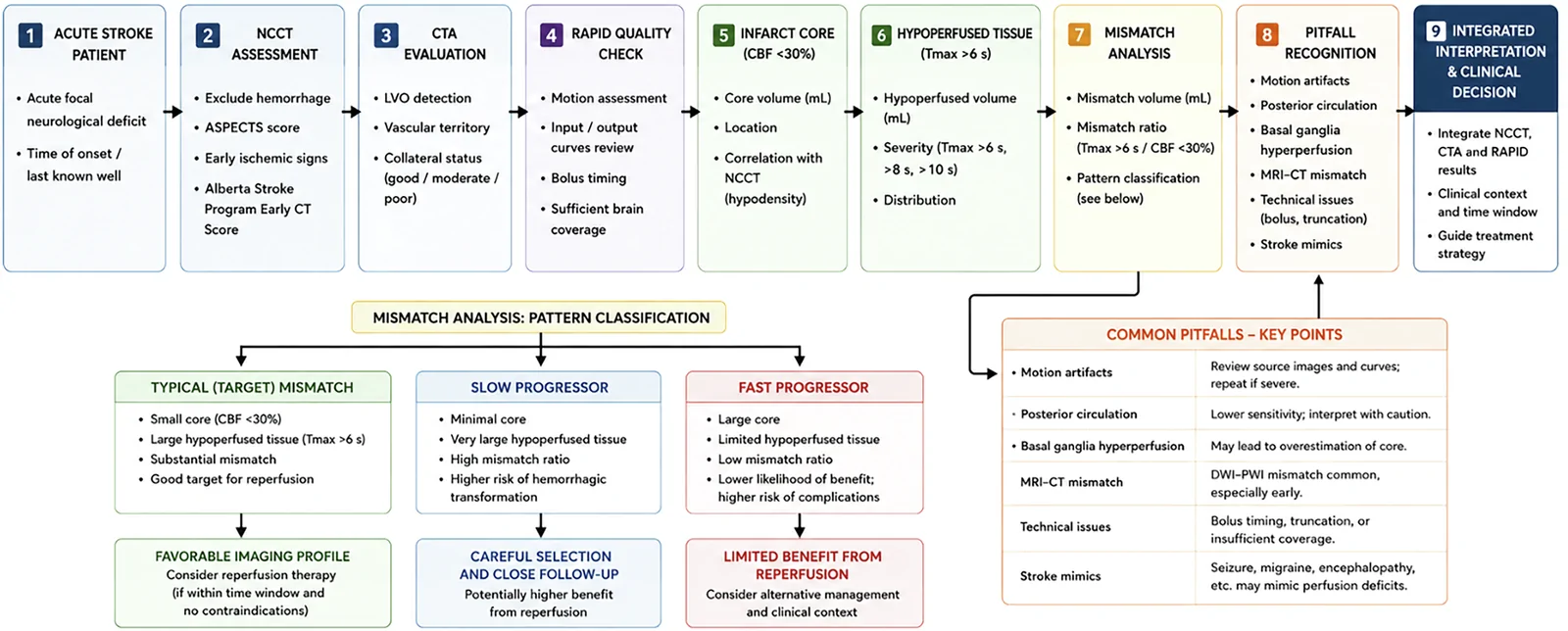
Practical workflow for RAPID CT perfusion interpretation in acute ischemic stroke. Interpretation should begin with NCCT and CTA assessment, followed by verification of RAPID data quality, evaluation of infarct core and hypoperfused tissue volumes, mismatch analysis, and systematic recognition of common diagnostic pitfalls. Final interpretation should integrate automated perfusion outputs with conventional imaging findings and clinical presentation

A structured approach to RAPID interpretation is summarized in Fig. 1 and integrates NCCT findings, vascular imaging, perfusion parameters, image quality assessment, and recognition of common diagnostic pitfalls.

To demonstrate how this structured diagnostic workflow applies to daily practice, the following sections present representative clinical scenarios illustrating classic perfusion profiles, subtle variants, and common diagnostic traps.

## Technical and algorithmic limitations of RAPID

Beyond transient artifacts and pitfalls, RAPID operates under intrinsic algorithmic limitations. Fixed hemodynamic cutoffs (CBF < 30% and *T*_max_ > 6 s) may not reflect the full biological continuum of ischemic injury in all patients. Furthermore, software performance remains limited in posterior fossa ischemia due to restricted Z-axis coverage and beam-hardening artifacts, as well as in small lacunar or deep gray matter infarcts where partial volume averaging obscures true perfusion deficits.

## Imaging patterns and diagnostic pitfalls

The principal RAPID-derived perfusion patterns, their clinical significance, and the most relevant interpretative pitfalls are summarized in Table 1. These imaging profiles provide a practical framework for understanding the representative examples illustrated in the following sections, bridging the gap between theoretical principles and daily clinical practice.

**Table 1:** Common RAPID-derived CT perfusion patterns, their typical imaging characteristics, clinical significance, and major interpretative pitfalls in acute ischemic stroke

Imaging pattern	Typical RAPID findings	Clinical interpretation	Potential pitfalls
Typical target mismatch	Small infarct core (CBF < 30%) with large *T*_max_ > 6 s lesion and high mismatch ratio	Large volume of potentially salvageable tissue; favorable candidate for reperfusion therapies	Automated volumes should always be correlated with NCCT and CTA findings
Slow progressor phenotype	Minimal or absent infarct core despite extensive *T*_max_ abnormality	Robust collateral circulation and delayed infarct progression; favorable tissue viability even in extended time windows	Hypoperfused volume may be overestimated when lower *T*_max_ thresholds are considered
Fast progressor phenotype	Large infarct core with limited mismatch volume	Advanced ischemic injury and reduced amount of salvageable tissue; generally associated with poorer prognosis	Core estimation may still be influenced by timing of imaging, reperfusion status, and technical factors
Intermediate mismatch profile	Moderate infarct core and moderate hypoperfused tissue volume	Requires individualized interpretation integrating symptoms, vessel status, collateral circulation, and treatment window	Mismatch ratio alone may not adequately reflect clinical relevance
Threshold-dependent *T*_max_ variation	Progressive increase in lesion volume at lower *T*_max_ thresholds (e.g., > 4 s) and reduction at higher thresholds (e.g., > 10 s)	Demonstrates the continuum of perfusion delay severity and collateral compensation	Lower thresholds may include benign oligemia, whereas higher thresholds identify more severe hypoperfusion
Motion-related artifact	Inconsistent, patchy, or non-vascular perfusion abnormalities; unreliable automated measurements	Technical limitation that may mimic or mask ischemic injury	Failure to review source images and motion-correction graphs may lead to diagnostic errors
CTP–MRI discordance	Disagreement between perfusion findings and subsequent DWI-MRI results	Reflects dynamic ischemic evolution, spontaneous reperfusion, or technical limitations	Automated maps should not be interpreted as an absolute representation of final infarct volume
Basal ganglia limitation	Small deep infarcts may not reach automated CBF or *T*_max_ thresholds	Reduced sensitivity of automated perfusion analysis in deep gray matter structures	High vascular density and small lesion volume may cause false-negative results
Posterior circulation limitation	Negative or subtle perfusion abnormalities despite clinically significant ischemia	Limited sensitivity of CTP in posterior fossa ischemia	Beam-hardening artifacts, lower signal-to-noise ratio, and limited coverage may reduce diagnostic accuracy
Stroke mimics	Absence of classical mismatch profile; occasional atypical perfusion abnormalities	Alternative neurological diagnosis should be considered (e.g., seizure, migraine, metabolic disorders)	Non-ischemic perfusion changes may be misinterpreted as acute stroke

The table summarizes the principal perfusion profiles encountered in daily practice and highlights the importance of integrating automated RAPID outputs with conventional imaging findings and clinical information

## The therapeutic “Sweet spot”—classical target core–penumbra mismatch

**Fig. 2:**
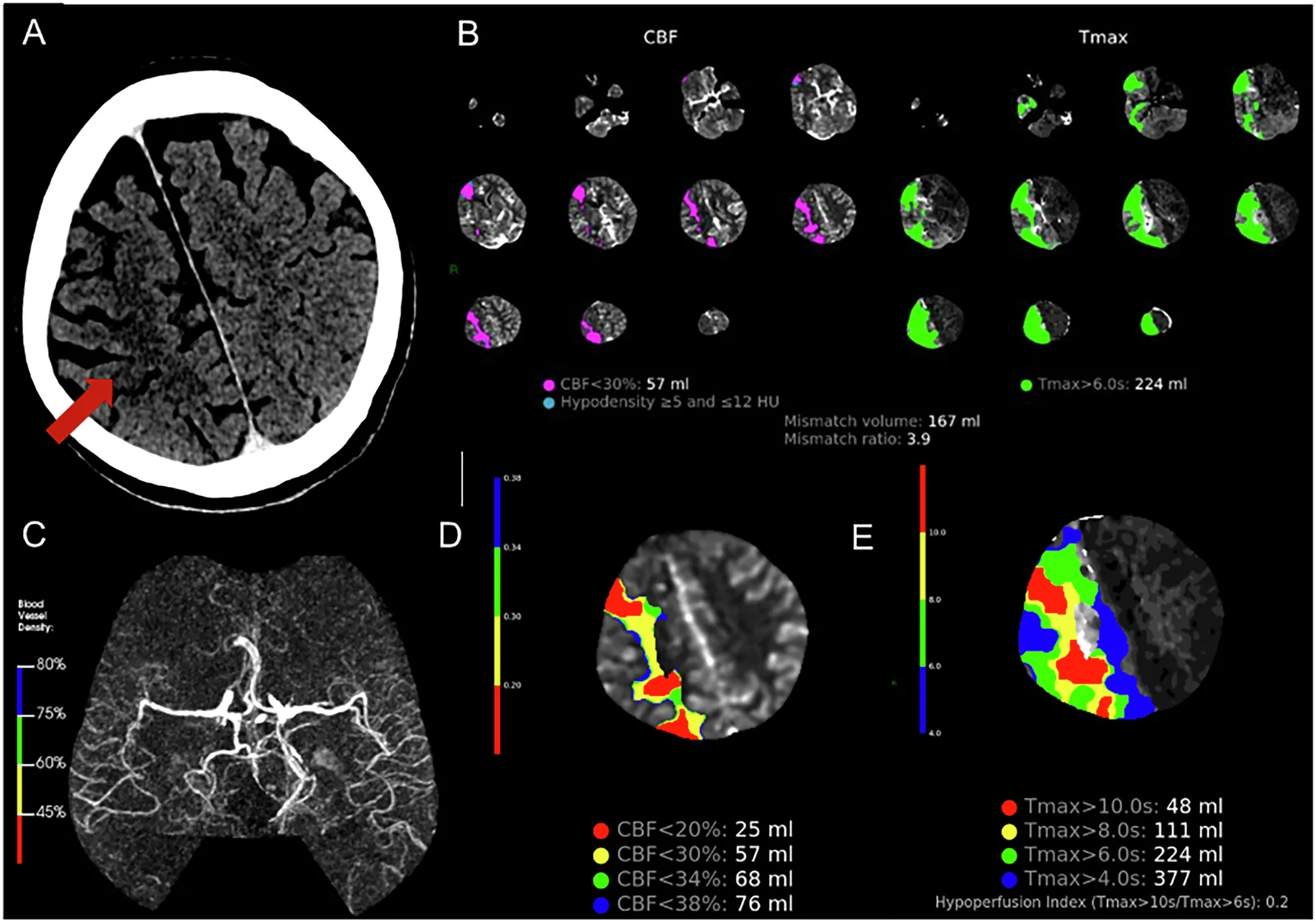
The therapeutic “Sweet Spot”—classic target mismatch. Representative example of an acute large vessel occlusion with a favorable core–penumbra mismatch profile, characterized by a relatively small infarct core and a large volume of potentially salvageable tissue, in a 70-year-old patient presented with nausea, headache, and left-sided hemiparesis. **A** Axial NCCT image at the level of the upper convexities reveals subtle ischemic changes, with only mild hypodensity noted at the level of the cerebral convexities of the left hemisphere. **B** CBF (CBF < 30%) and *T*_max_ maps demonstrate a significant core-penumbra mismatch. Note the core volume of 57 mL and a vast hypoperfused territory of 224 mL, resulting in a favorable mismatch ratio of 3.9. This pattern confirms substantial potentially salvageable tissue. **C** CTA maximum intensity projection (MIP) shows an arterial density map overlaid, illustrating the perfusion deficit location. **D** Multithreshold CBF analysis reveals that while the core definition changes, the automated calculation for < 30% aligns well with the visible signs on NCCT, pointing towards established infarction. **E** Multithreshold *T*_max_ analysis displays stratification across increasing delay thresholds, suggesting progressive severity of perfusion delay in the affected hemisphere (nesting appearance). The calculated hypoperfusion index of 0.2 indicates favorable collaterals and preserved penumbra

The prototypical pattern of perfusion in acute ischemic stroke is the presence of a large mismatch ratio, sustained by a small infarct core with an extensive region of penumbra with delayed perfusion (Fig. 2). This mismatch profile confirms the presence of at-risk, but not yet irreversibly damaged tissue (penumbra), and is therefore one of the most clinically relevant situations for acute stroke imaging. In fact, it represents the biological substrate for treatment responsiveness established by DEFUSE and DAWN trials for reperfusion therapy [4, 5, 8]. In this scenario, NCCT may reveal only minor early ischemic changes, while a CBF map identifies a small core and the *T*_max_ reveals a greater perfusion deficit encompassing a larger vascular territory, confirming a high volume of salvageable tissue.

## The “slow progressor”—large mismatch with minimal infarct core

**Fig. 3:**
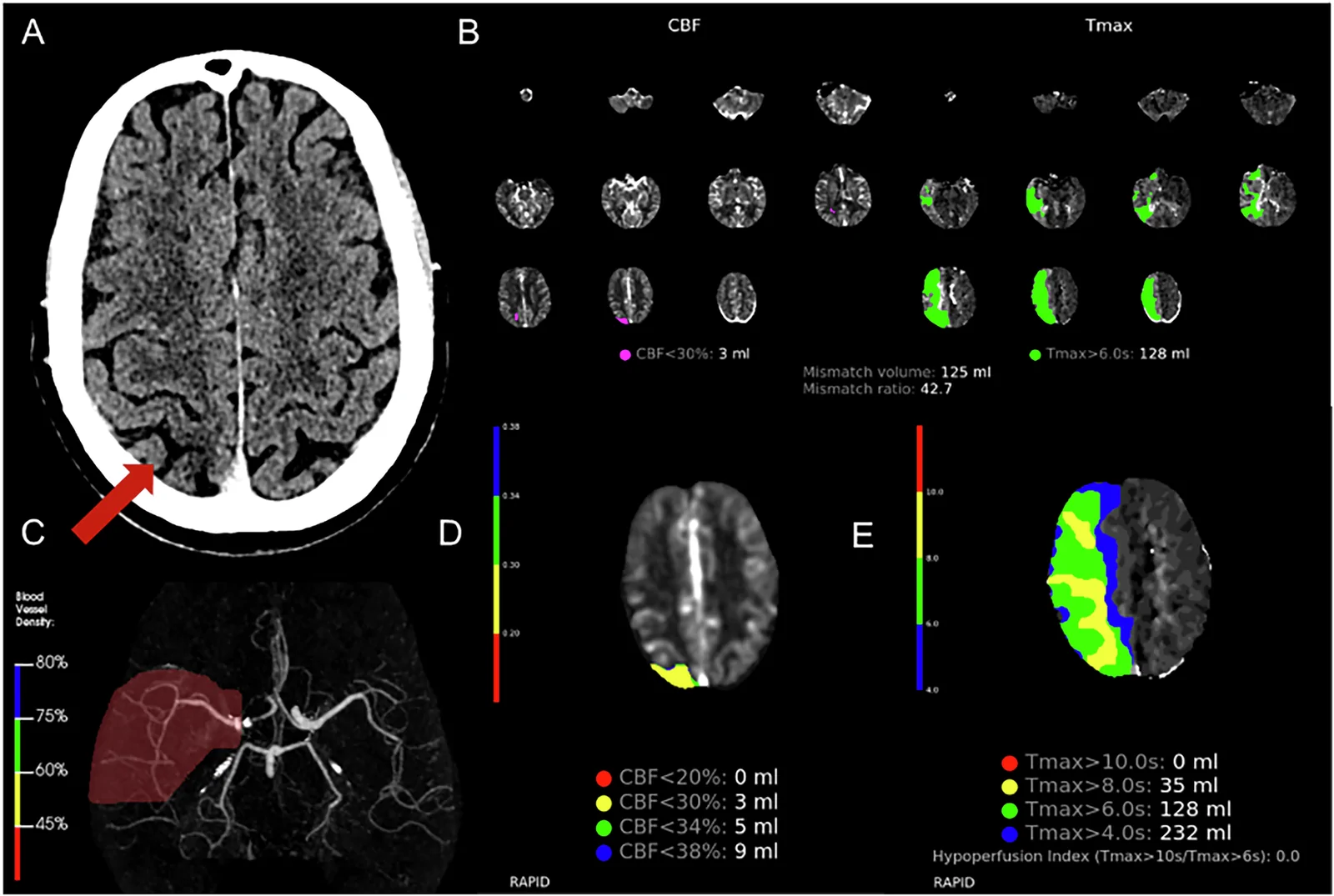
The “Slow Progressor”—large mismatch with minimal infarct core. Representative example of a slow progressor phenotype, showing extensive hypoperfused tissue despite a negligible infarct core, consistent with robust collateral circulation and prolonged tissue viability, in an 81-year-old female patient presented with a left-sided motor hemisyndrome, nausea, and severe headache. **A** Axial NCCT at the level of the upper convexities demonstrates a normal parenchymal appearance (ASPECTS 10) despite the acute clinical presentation. The red arrow highlights the territory at risk, which remains morphologically intact on baseline imaging. **B** CBF (CBF < 30%) and *T*_max_ maps reveal a striking mismatch profile. The cerebral blood flow map (CBF < 30%) identifies a negligible infarct core of only 3 mL, while the *T*_max_ map (> 6.0 s) demonstrates a massive 128 mL hypoperfused territory. The resulting mismatch ratio of 42.7 is indicative of an “extreme” slow progressor phenotype. **C** CTA MIP confirms an acute occlusion of the right M1 segment of the middle cerebral artery (MCA), with the red overlay indicating the corresponding downstream vascular territory under critical hypoperfusion. **D** Multithreshold CBF analysis confirms the stability of the core volume across various thresholds (CBF < 20% to < 38%), with 0 mL at the most severe threshold (< 20%), suggesting robust collateral support that prevents rapid tissue transition to irreversible injury. **E** Multithreshold *T*_max_ analysis displays the “stratified” delay thresholds (*T*_max_ > 4 s to > 10 s). Notably, the Hypoperfusion Index (*T*_max_ > 10 s/*T*_max_ > 6 s) is 0.0, indicating that while a large territory is delayed, none of the tissue has yet reached the threshold of critical hypoperfusion. This profile represents the ideal candidate for late window thrombectomy

In some patients, the infarct core remains remarkably small or absent, whereas the *T*_max_ abnormality is extensive, despite a high-grade arterial occlusion (Fig. 3). This situation reflects a very an extremely high mismatch ratio and is consistent with robust collateral circulation that prevents or significantly delays the conversion of penumbra into irreversible infarction [14]. These patients represent the ideal candidates for treatment in extended time windows, as the brain remains in a state of sustained viability, with a large amount of salvageable tissue despite major hypoperfusion.

## The “Fast Progressor”—large infarct core without significant mismatch

**Fig. 4:**
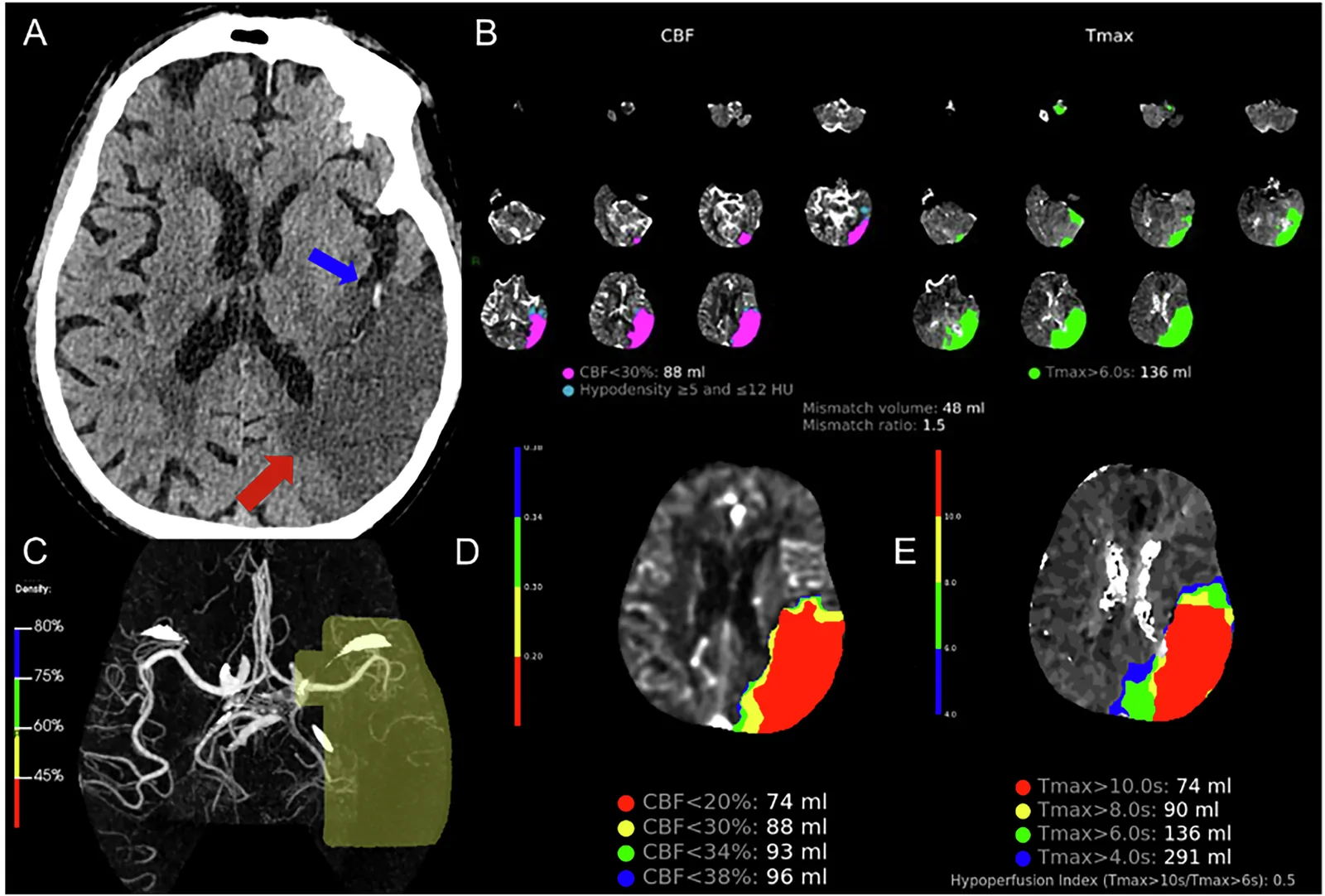
The “Fast Progressor”—large infarct core with malignant profile. Representative example of a fast progressor phenotype, characterized by extensive infarct core formation, limited salvageable tissue, and poor collateral compensation, in a 94-year-old male patient presented with global aphasia, dysarthria, right-sided hemiparesis, and sphincteric release. **A** Axial NCCT at the level of the basal ganglia reveals extensive, well-defined hypodensity involving the left MCA territory (red arrow), associated with loss of the insular ribbon and subtle mass effect. A hyperdense MCA sign (blue arrow) is visible, indicating acute occlusion due to thromboembolism. **B** CBF (CBF < 30%) and *T*_max_ maps demonstrate a “malignant” perfusion profile. The cerebral blood flow map (CBF < 30%) shows a large established infarct core of 88 mL. The *T*_max_ map (> 6.0 s) reveals a total hypoperfused volume of 136 mL, resulting in a low mismatch ratio of 1.5. This pattern indicates that the majority of the affected territory has already transitioned to irreversible injury. **C** CTA MIP confirms a proximal occlusion of the left M1 segment, with the yellow overlay highlighting the broad vascular territory involved. **D** Multithreshold CBF analysis demonstrates that a substantial portion of the lesion is already below the critical threshold of CBF < 20% (74 mL), confirming dense ischemia. This profile is associated with a higher risk of hemorrhagic transformation and limited potential for clinical benefits from late-window reperfusion. **E** Multithreshold *T*_max_ analysis shows a high Hypoperfusion Index (*T*_max_ > 10 s/*T*_max_ > 6 s) of 0.5. The large volume of critically delayed tissue (*T*_max_ > 10 s = 74 mL, red) is nearly equivalent to the infarct core, suggesting poor collateral compensation and rapid ischemic recruitment

At the opposite end of the spectrum, some patients exhibit a large infarct core represented by extensive reductions in CBF, with only minor increases in *T*_max_ beyond the infarct region representing the penumbra, and thus a low mismatch ratio (Fig. 4).

This pattern indicates rapid ischemic recruitment often due to poor collateral status, with advanced irreversible injury and only a small amount of potentially salvageable tissue. While recent trials (SELECT2, TENSION) suggest some large-core patients may still benefit, this phenotype is usually associated with poorer prognosis and reduced potential benefit from reperfusion treatment, also due to a higher risk of hemorrhagic transformation. In addition, recent results have broadened the discussion for some patients with large infarct cores [17].

## The impact of *T*_max_ threshold stratification on penumbral estimation

The interpretation of automated perfusion maps is closely related to the hemodynamic thresholds applied during post-processing. Variation in *T*_max_ cutoffs may lead to significant discrepancies in the estimated volume of hypoperfused tissue. While permissive thresholds (e.g., *T*_max_ > 4 s) may encompass areas of benign oligoemia, thereby overestimating the volume of tissue at risk, more stringent thresholds (*T*_max_ > 10 s) isolate regions of critical hypoperfusion and are often indicative of failing collateral circulation [2, 3, 14].

**Fig. 5:**
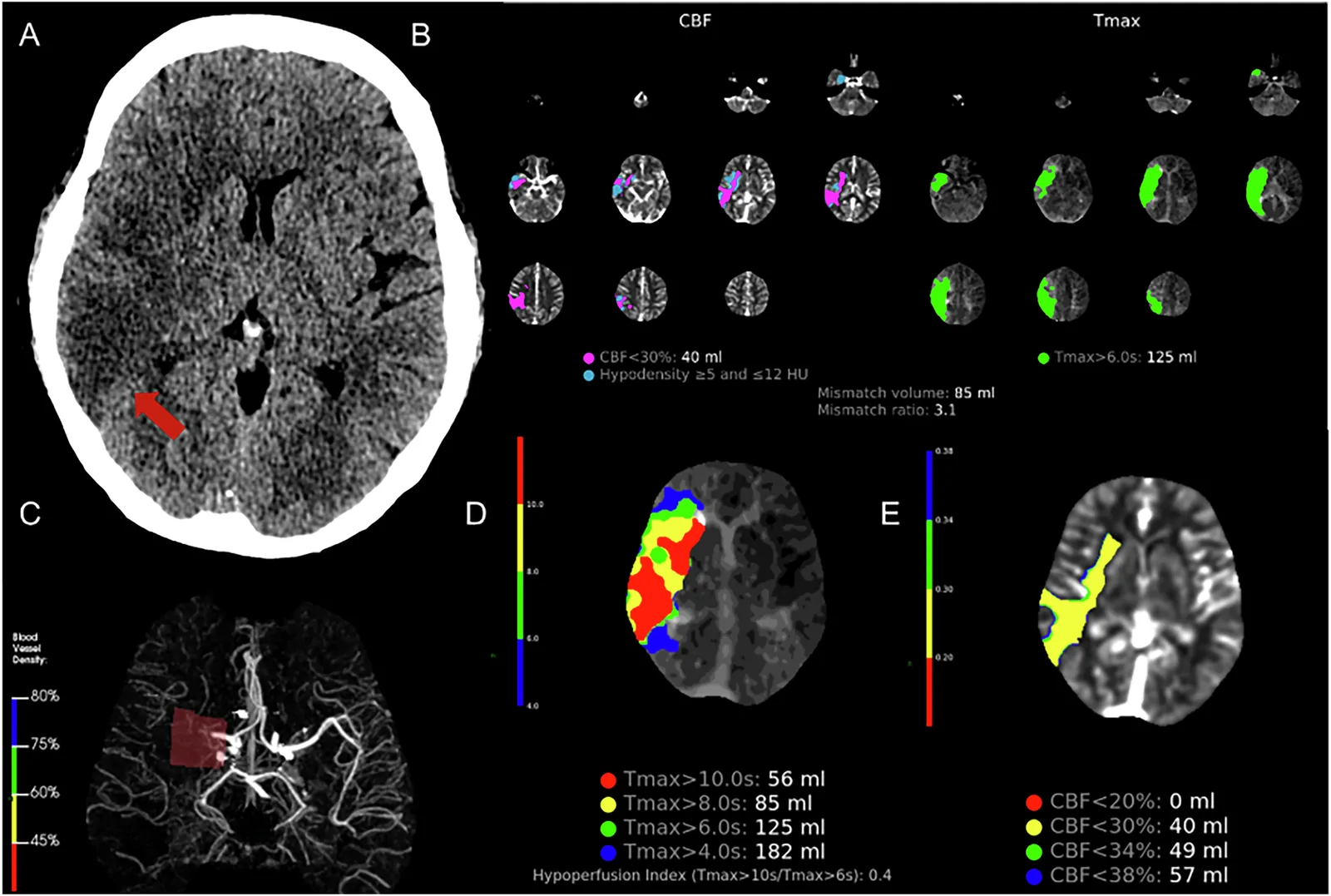
The threshold effect—navigating *T*_max_ and CBF Sensitivity. Representative example illustrating the influence of different *T*_max_ thresholds on the estimation of hypoperfused tissue volume and the interpretation of penumbral extent, in a 61-year-old female presented with acute neurological deficits. **A** Axial NCCT at the level of the basal ganglia reveals established ischemic changes in the left MCA territory (red arrow) and a prominent hyperdense MCA sign (blue arrow), indicative of acute large vessel occlusion. **B** CBF (CBF < 30%) and *T*_max_ maps using standard thresholds ($CBF$ < 30% and *T*_max_ > 6 s). The automated summary identifies a 40 mL core and a 125 mL hypoperfused territory, yielding a mismatch ratio of 3.1. **C** CTA MIP demonstrates the left M1 segment occlusion, with the red overlay highlighting the downstream perfusion deficit. **D** Multithreshold *T*_max_ analysis illustrates the “nested” stratification of perfusion delay. Note how the abnormal volume expands to 182 mL when using the most sensitive threshold (*T*_max_ > 4 s, blue). The Hypoperfusion Index (*T*_max_ > 10 s/*T*_max_ > 6 s) of 0.4 indicates that nearly half of the penumbra is under critical hypoperfusion (*T*_max_ >10 s = 56 mL, red), a marker of moderate collateral failure. **E** Multithreshold CBF analysis demonstrates that while 40 mL of tissue meets the CBF < 30% criteria, 0 mL is currently below the more severe CBF < 20% threshold. This discrepancy suggests that although an infarct core is present, the degree of flow reduction has not yet reached the most critical levels across the entire lesion, potentially defining a “gray zone” for therapeutic timing

This technical specificity is essential because automated software appears to provide an impression of objective accuracy but results in maps that are still dependent on the choice of thresholds. Fig. 5 illustrates this sensitivity, demonstrating progressive volumetric shifts across different *T*_max_ thresholds with no significant change in the volume of the CBF-defined core.

## The “Warning Zone”—intermediate perfusion patterns (moderate mismatch)

A large proportion of patients do not conform to either of the clearly favorable or unfavorable profiles outlined above but instead exhibit intermediate perfusion profiles with moderate infarct core and variable peripheral hypoperfusion. These cases are among the most challenging encountered in routine practice, as the automated mismatch metrics may hover near the traditional inclusion thresholds. In this scenario, the decision for reperfusion therapy cannot rely solely on the software output but must also integrate other parameters such as severity of symptoms, location of vascular occlusion, involvement of eloquent areas, status of collateral perfusion, age, time of onset, and perfusion parameters [13].

**Fig. 6:**
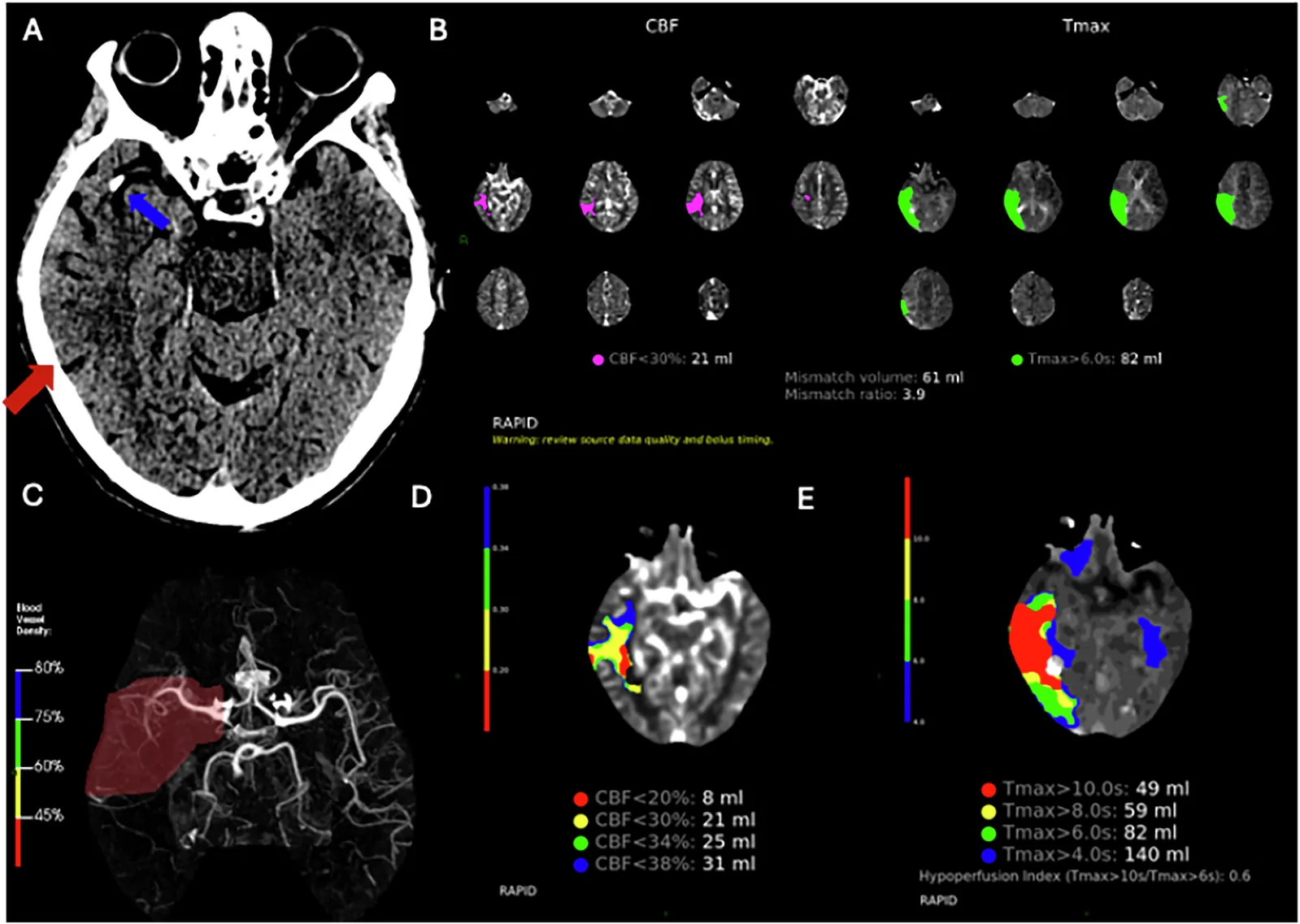
The “Warning Zone”—integrating technical alerts with clinical reality. Representative example of an intermediate perfusion pattern, highlighting the need for integrated interpretation of perfusion parameters, vascular findings, collateral status, and clinical presentation, in an 85-year-old female who presented with a sudden onset of motor deficit involving the left hand, which rapidly progressed to a complete left-sided motor hemisyndrome. **A** Axial NCCT at the level of the sylvian fissure shows early ischemic signs, including subtle loss of the insular ribbon and cortical swelling (red arrow). A hyperdense MCA sign is present (blue arrow). **B** CBF (CBF < 30%) and *T*_max_ maps indicate a mismatch profile with a 21 mL core (CBF < 30%) and 82 mL of hypoperfused tissue (*T*_max_ > 6 s). Notably, the software generates a yellow warning regarding source data quality and bolus timing. This alert necessitates a meticulous review of the concentration-time curves to exclude truncation or suboptimal contrast arrival that could skew volumetric results. **C** CTA MIP confirms the right M2 occlusion, with the red overlay delineating the threatened vascular territory. **D** Multithreshold CBF analysis shows a core volume that remains relatively stable between the < 20% (8 mL) and < 30% (21 mL) thresholds, suggesting a clearly defined area of dense ischemia. **E** Multithreshold *T*_max_ analysis reveals a high Hypoperfusion Index (*T*_max_ > 10 s/*T*_max_ > 6 s) of 0.6. More than half of the penumbra is already critically delayed (*T*_max_ > 10 s = 49 mL, red), signaling poor collateral recruitment and a high risk of rapid infarct expansion despite the seemingly “favorable” mismatch ratio of 3.9. This case highlights how the Hypoperfusion Index can provide a more accurate physiological snapshot than summary volumes alone

This case (Fig. 6) underscores a fundamental tenet of modern stroke imaging: while RAPID provides the data, the radiologist needs to combine imaging results with the overall clinical situation. Treatment must be tailored to the patient’s unique clinical trajectory rather than dictated by a rigid, automated numerical cutoff.

## Diagnostic traps—motion artifacts and technical mimics

Despite the sophistication of automated algorithms, image quality remains the ultimate arbiter of diagnostic reliability. Motion artifacts are the “Achilles’ heel” of CTP, being among the most common causes of misleading maps with the generation of patchy, non-physiological perfusion defects that can mimic or exaggerate a true ischemic penumbra. These “pseudo-mismatch” patterns often manifest as irregular, curvilinear abnormalities that ignore established vascular territories. In addition, technical factors including poor timing of contrast bolus, incomplete acquisition, and noise influence both visual maps and numerical results [7, 10].

**Fig. 7:**
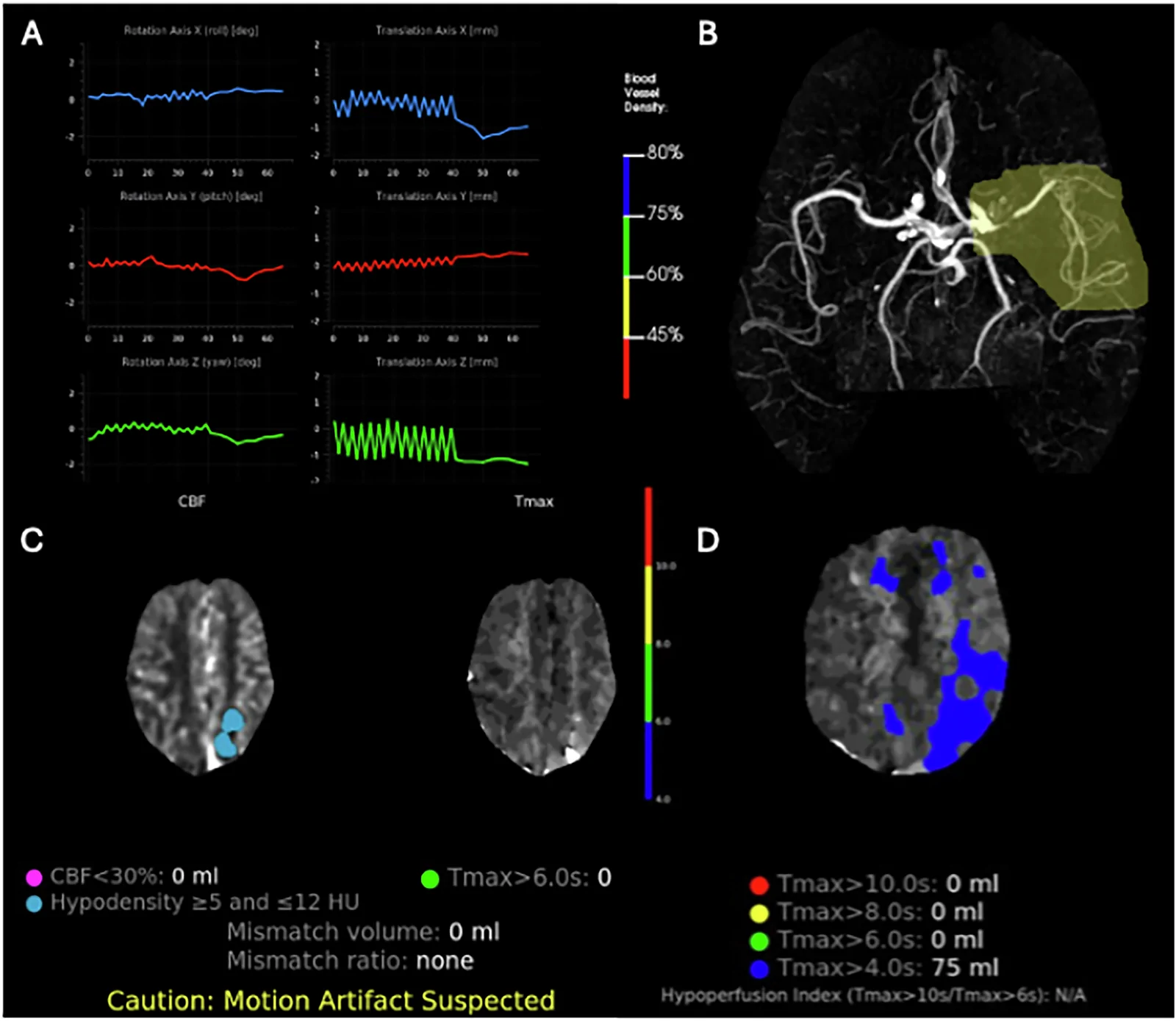
Motion artifacts leading to core underestimation. Representative example demonstrating the impact of patient motion on RAPID post-processing, resulting in unreliable core estimation and potentially misleading perfusion maps, in a 79-year-old male patient who presented with an acute onset of symptoms, such as right-sided hemiparesis, inability to walk independently, flattening of the right nasolabial fold, and complete plegia of the right upper and lower limbs. **A** Graphs of rotation and translation: confirm patient motion, represented by multiple sharp spikes in the rotation and translation axes. Such abrupt movements frequently distort the deconvolution algorithm, leading to unreliable volumetric data. **B** CTA MIP: shows an overlay indicating the area of concern at the level of the left MCA territory. **C** CBF (CBF < 30%) and *T*_max_ maps paradoxically indicate a 0 mL core (CBF < 30%). This represents a critical technical pitfall: motion-induced noise can mask established ischemic changes, leading to a failure in core identification even when frank hypodensity is visible on NCCT. **D** *T*_max_ map: threshold stratification is key. The automated map shows extensive *T*_max_ > 4 s hypoperfusion (75 mL, blue), but 0 mL for all higher thresholds (*T*_max_ > 6 s, > 8 s, > 10 s). In the context of significant motion, this discrepancy—where only the most permissive threshold is triggered—further suggests that the automated output is a technical artifact rather than a true physiological representation

Critically, motion-induced noise not only creates false positives; it can also lead to a dangerous underestimation of the infarct core. As illustrated in Fig. 7, abrupt patient movement can distort the deconvolution process to the point of masking established ischemic changes. This may result in an automated “zero-core” report (CBF < 30% = 0 mL) that paradoxically contradicts visible parenchymal hypodensity on NCCT.

Reliance on automated summary maps without a meticulous review of the raw source images and the motion-correction graphs is a recipe for diagnostic error. This case serves as a critical reminder: artifact recognition is a fundamental radiological competency that prevents “automation bias” from overriding clinical and morphological reality.

## Discrepancy between CTP and MRI

While automated CT perfusion analysis provides vital guidance during the hyperacute triage of stroke, its findings do not always align with subsequent MRI. Recognizing the pathophysiological and timing-related reasons for CTP–MRI discordance is essential to avoid misinterpreting transient, evolving, or technical perfusion changes. For instance, a focal CTP abnormality may not always translate into restricted diffusion on MRI; such discrepancies often reflect transient ischemic attacks (TIA), early spontaneous reperfusion, or technical overestimation of the ischemic burden [9, 12, 18]. Conversely, the “ghost infarct core” phenomenon—where a large CBF-defined core on CTP significantly overestimates the final infarct volume seen on MRI—remains a critical pitfall in hyperacute imaging.

**Fig. 8:**
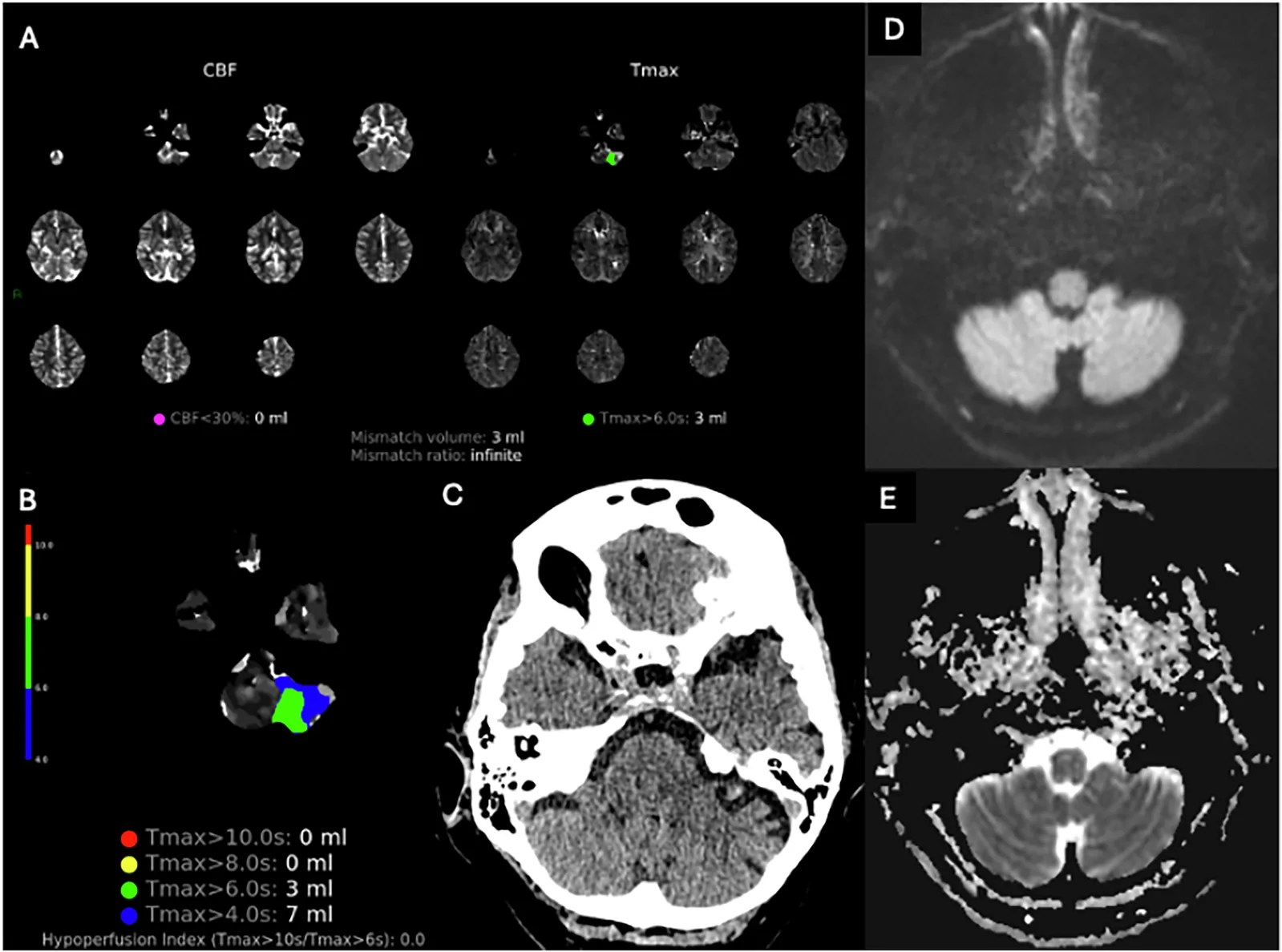
Modality discordance. Representative example illustrating the potential discrepancy between CT perfusion findings and subsequent MRI results, emphasizing the dynamic nature of ischemic injury and the limitations of automated thresholds, in a 70-year-old female presented with asthenia, left-sided oral commissure deviation, and reported balance impairment. **A**, **B** CBF (CBF < 30%) and *T*_max_ maps and multithreshold *T*_max_ maps show negligible perfusion delay (*T*_max_ maps > 6 s = 3 mL), providing a false sense of security. **C** Axial NCCT demonstrates no clear hypodensity. **D**, **E** DWI and ADC maps obtained shortly after do not reveal focal areas of restricted diffusion in the left cerebellar hemisphere. This case illustrates a different facet of the “modality gap”: when both CTP and DWI are negative in a symptomatic patient, the diagnosis often shifts toward a TIA or a “minor stroke” with rapid recanalization. It underscores that automated software and even gold-standard MRI can be negative if the ischemic insult is transient or below the threshold of detection, highlighting the vital importance of the initial clinical examination in guiding patient management and secondary prevention

This modality gap, illustrated in Fig. 8, underscores the importance of regarding automated perfusion software as a decision-support tool rather than an absolute truth. Variations in imaging timing and collateral dynamics can create a “moving target” effect, where the physiological state of the brain evolves between the CT and MRI acquisitions. Ultimately, these discrepancies reinforce the need for a multimodal diagnostic framework, where the radiologist adjudicates between conflicting data points to provide the most accurate clinical prognosis.

## Anatomical blind spots: the pitfalls of basal ganglia and posterior circulation

**Fig. 9:**
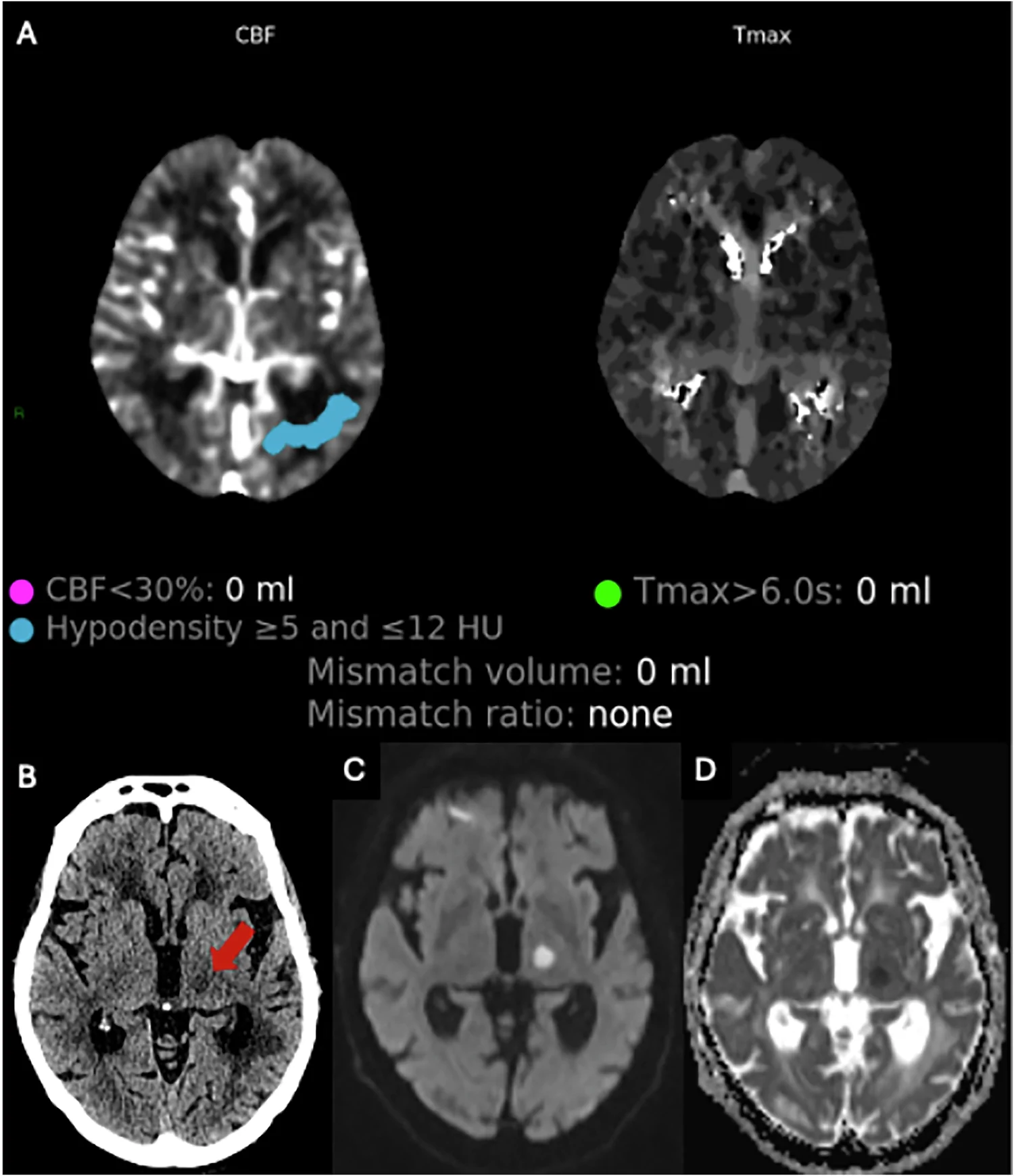
Anatomical blind spots: the pitfall of basal ganglia. Representative example demonstrating underestimation of ischemic injury within deep gray matter structures, where automated perfusion thresholds may fail to identify small but clinically significant infarcts, in a 92-year-old female patient presented with acute dysarthria, spatial and temporal disorientation, and a motor deficit (sthenic deficit) involving the right lower limb. **A** CBF (CBF < 30%) and *T*_max_ maps demonstrate a completely negative profile, with 0 mL calculated for both the infarct core (CBF < 30%) and hypoperfused territory (*T*_max_ > 6.0 s). **B** Axial NCCT shows a small hypointense area in the left thalamus (red arrow), not identified by RAPID (ASPECTS 10). **C**, **D** MRI diffusion-weighted imaging (DWI) and ADC Map confirm the presence of a focal area of restricted diffusion in the left thalamus, consistent with an acute infarct. This discrepancy exemplifies a major limitation of automated CTP: the “threshold effect” in deep gray matter. Because of high baseline vascularization and small lesion volume, these infarcts often do not trigger the CBF < 30% or *T*_max_ > 6 s criteria, necessitating a high clinical index of suspicion and gold-standard MRI correlation for definitive diagnosis

**Fig. 10:**
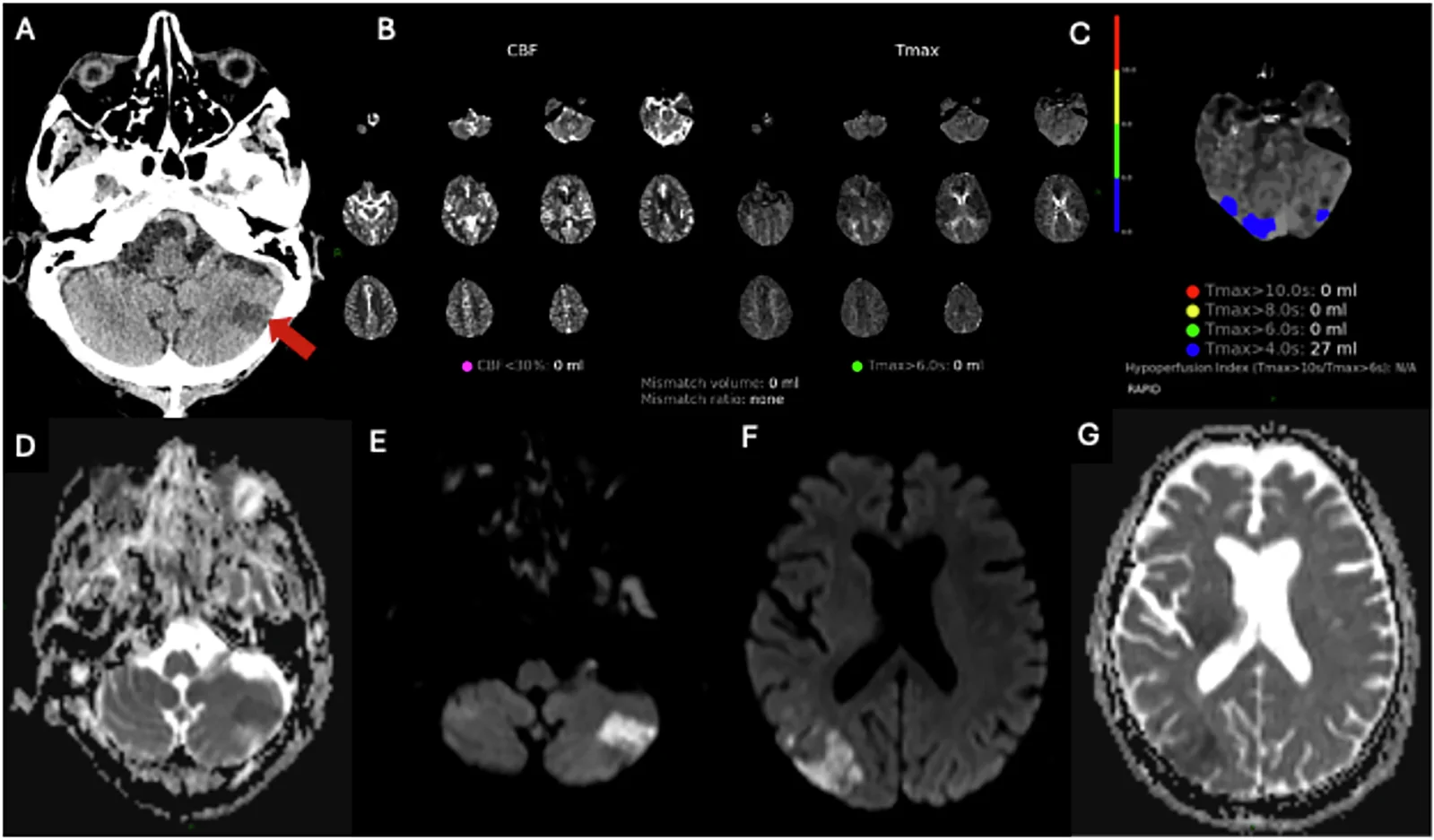
Anatomical blind spots: the pitfall of the posterior circulation. Representative example highlighting the limited sensitivity of CT perfusion in the posterior circulation, where technical and anatomical factors may lead to false-negative RAPID results, in a 70-year-old male patient presented with acute sensory disturbances of the face and an evident deviation of the oral commissure. **A** Axial NCCT reveals a subtle but definitive area of low attenuation in the left cerebellar hemisphere (red arrow), though visualization is often hampered by bone-induced artifacts in this region. **B** CBF (CBF < 30%) and *T*_max_ maps demonstrate a complete diagnostic failure of the automated software. Both the infarct core (CBF < 30%) and the hypoperfusion territory (*T*_max_ > 6.0 s) are reported as 0 mL. This “false negative” is a known limitation in the posterior circulation due to lower signal-to-noise ratios and unique hemodynamics. **C** Multithreshold *T*_max_ analysis shows only a minimal, non-specific volume (27 mL) at the most permissive threshold (*T*_max_ > 4.0 s, blue). All clinical thresholds (*T*_max_ > 6 s, > 8 s, > 10 s) are entirely negative. **D**, **E** MRI DWI and ADC Map (infratentorial) confirm an acute infarct in the left cerebellar hemisphere with marked restricted diffusion, correlating with the NCCT findings but completely missed by CTP. **F**, **G** MRI DWI and ADC map (supratentorial) demonstrate another focus in the right temporal-parietal region. This multi-territorial infarct pattern, combined with the CTP failure, underscores that in the posterior circulation, clinical judgment and MRI must override automated “zero-volume” reports to avoid catastrophic undertreatment

In addition to patient-related and technical factors, automated post-processing software encounters physiological and anatomical constraints in specific cerebral regions. In fact, while RAPID offers robust quantification for cortical middle cerebral artery (MCA) territories, its reliability significantly diminishes in specific anatomical regions—most notably the basal ganglia (Fig. 9) and the posterior circulation (Fig. 10).

Infarct core estimation in deep gray matter is inherently confounded by high baseline attenuation, dense vascularization, and susceptibility to partial volume effects. These factors often render the fixed CBF < 30% threshold unreliable, potentially leading to spurious volumetric assessments in the hyperacute phase [7, 18].

The posterior fossa represents an even greater diagnostic challenge, where technical and physiological constraints converge. The brainstem and cerebellum are frequently affected by limited *Z*-axis coverage, beam-hardening artifacts from the petrous bone, and a lower signal-to-noise ratio. Furthermore, vertebrobasilar ischemia may produce only marginal *T*_max_ delays, even in clinically catastrophic presentations, making the thresholds derived from anterior circulation data largely inapplicable [19–21].

As illustrated in this final composite analysis, a “negative” or “subtle” RAPID map in these territories must never override clinical judgment. When ischemia is suspected in the basal ganglia or posterior fossa, the radiologist must pivot to a multimodal integration of NCCT, CTA, and clinical findings. In these anatomical blind spots, MRI with DWI remains the indispensable arbiter, as CTP—and by extension RAPID—is prone to significant underestimation of the true ischemic burden [20].

## Stroke mimickers

Stroke mimickers make up nearly half of suspected stroke admissions. They can present as functional disorders or symptoms of neurological or medical conditions. Despite careful assessment, many patients still receive unnecessary thrombolysis and intensive care. Accurate diagnosis is crucial, as recurrences are common [22]. Accurate diagnosis is important. CT perfusion could help in these events also when the results of software are negative.

**Fig. 11:**
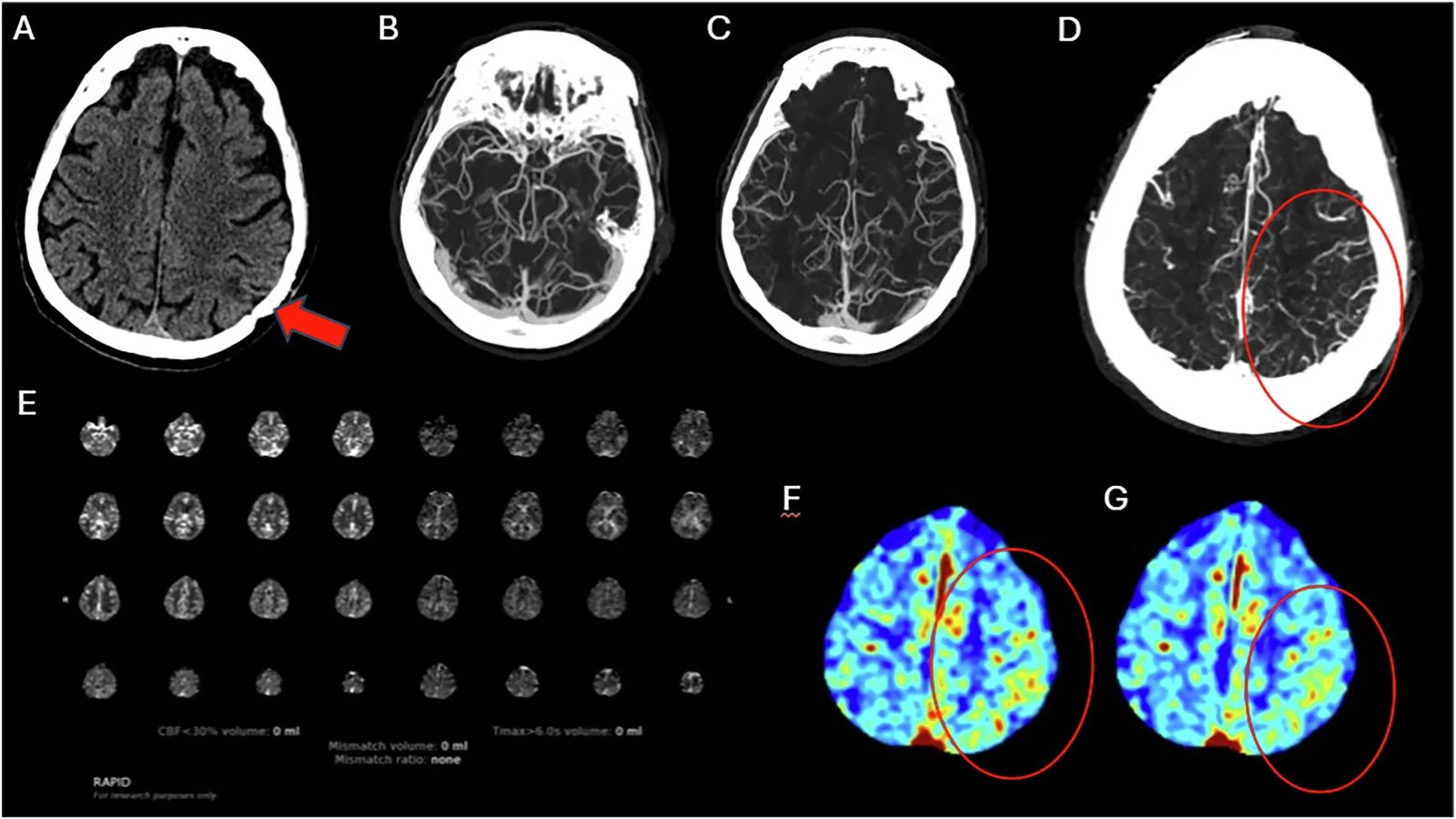
Stroke mimics. Representative example of a stroke mimic in which conventional RAPID mismatch parameters remain negative despite abnormal perfusion findings suggestive of an alternative neurological diagnosis, in an 83-year-old woman who presents to the emergency room in a state of confusion and with alien hand syndrome on the right. **A** Axial NCCT shows the craniotomy hole in the left parietal area and a small area of cerebral swelling in the underlying cortex (red arrow). **B**–**D** CTA MIP does not show arterial vascular obstructions, but at the left vertex (**D**) congestion of arterial vessels is appreciated, more evident than on the contralateral side (red circle). **E** CBF (CBF < 30%) and *T*_max_ maps do not reveal any alterations in CBF or *T*_max_ maps relative to reference parameters for the presence of an ischemic “core” or penumbra. **F**–**G** Cerebral blood volume (**F**) and CBF (**G**) colorimetric maps, however, confirm an abnormal increase in parameters in the left parietal region (red circle), suggesting a critical condition, which was later confirmed by electroencephalographic study

Undetected seizure with post-ictal deficits and a Todd’s paresis, in which the patient presents with transient postictal focal weakness, is one of the most common presentations of a stroke mimic. Less commonly, a focal atonic seizure in which the ictal feature is transient focal weakness, typically lasting for a few seconds, can be misdiagnosed as a TIA. Further complicating the diagnosis, seizures can also be the initial manifestation of an acute stroke (Fig. 11).

## Discussion

CTP analyzed with RAPID is now an essential component of modern stroke imaging and has contributed greatly to the shift from a rigid time-based treatment strategy to a tissue-clock paradigm. This evolution is grounded in landmark randomized trials demonstrating superior outcomes for patients selected via clinical-imaging mismatch and perfusion-based tissue viability, extending the therapeutic window for thrombectomy and thrombolysis up to 24 and 9 h, respectively [8].

Extensive literature evaluates the diagnostic performance of RAPID CTP, demonstrating high sensitivity (85–92%) and specificity (88–95%) for identifying core and penumbra when compared to expert radiological readings and follow-up MRI DWI. While its automated algorithms offer high inter-software reproducibility, diagnostic variability can still arise from patient-specific parameters such as contrast delivery dynamics, severe motion, and baseline vessel tortuosity [8].

Technically, CBF less than 30% represents one of the most widely accepted thresholds for estimating the infarct core. [2, 3, 23]. However, this threshold does not represent absolute biological truth, but it is more of an operational definition, based on validation studies. This metric is influenced by acquisition protocol, scanner characteristics, method of deconvolution, and patient hemodynamics [7]. Consequently, quantitative outputs must be contextualized within a broader radiological framework.

The progression of an infarct is fundamentally determined by the status of collateral circulation, which is indirectly reflected in RAPID variables, including *T*_max_ delay and the hypoperfusion index [14]. Although RAPID provides standardized results, automated software may produce a “false sense of certainty” if technical confounders, such as motion artifacts, contrast bolus kinetics, or truncated acquisitions, are not meticulously scrutinized [10, 18].

A pivotal evolution in acute stroke management involves the treatment of patients with large infarct cores. Traditionally viewed as a contraindication to intervention, recent trials (e.g., SELECT2, TENSION) indicate that selected patients with extensive baseline injury may still experience significant clinical benefit from mechanical thrombectomy [17].

This emerging evidence challenges rigid exclusion based solely on a numerical volume, emphasizing that the “salvageability” of tissue is a dynamic biological continuum rather than a binary metric [24, 25].

Variations in quantitative estimates across different commercial software packages stem primarily from differences in mathematical deconvolution algorithms (e.g., delay-insensitive vs standard singular value decomposition), automated selection criteria for arterial input functions (AIF) and venous output functions (VOF), and subtle differences in thresholding logic [15].

In clinical practice, these technical discrepancies can lead to significant volume shifts (up to 15–20 mL), potentially reclassifying patients near trial inclusion cutoffs (such as a 70 mL core cutoff) and altering treatment decisions. Therefore, the radiologist should always review raw source data, correlate perfusion abnormalities with NCCT and CTA findings, and integrate the overall clinical status.

Furthermore, recent trials and meta-analyses (such as PRACTISE) have questioned whether mandatory CTP offers a tangible clinical benefit over high-quality NCCT and CTA alone in early therapeutic windows or unselected patient populations. While CTP remains indispensable for late-window selection and wake-up strokes, relying exclusively on CTP may unnecessarily delay reperfusion or exclude eligible candidates in straightforward early-window scenarios.

Conversely, CTP remains a high-value tool in patients with mild clinical deficits (low NIHSS), where a “nontrivial” proportion may still harbor substantial at-risk tissue that warrants intervention.

## Conclusions

CT perfusion processed with RAPID software is a transformative decision-support tool designed to assist, rather than replace, the radiologist by streamlining the neuroradiological workflow, facilitating precise, tissue-based decision-making in acute stroke. By providing standardized hemodynamic metrics, it serves as a powerful adjunct for both radiologists and clinicians, particularly in extended therapeutic windows.

However, automated outputs must never be used as stand-alone criteria for treatment decisions, but should serve as objective data to be integrated into the broader clinical, morphological, and vascular picture under expert radiological oversight. Crucially, automated maps must be meticulously adjudicated to identify technical artifacts, threshold dependencies, and anatomical limitations. Ultimately, the most effective workflow synergizes technological speed with human diagnostic acumen, ensuring that therapeutic decisions remain firmly grounded in biological reality rather than algorithmic outputs. This review serves as a practical guide to mastering these imaging patterns, ultimately empowering neuroradiologists to minimize interpretive errors and deliver precise, individualized care for patients with acute ischemic stroke.

## Data Availability

The datasets analyzed during the current study (anonymized CT and MRI images) are available from the corresponding author on reasonable request, in compliance with institutional privacy regulations.

## Abbreviations

ADC: Apparent diffusion coefficient
ASPECTS: Alberta stroke program early CT score
CBF: Cerebral blood flow
CTA: Computed tomography angiography
CTP: Computed tomography perfusion
DWI: Diffusion-weighted imaging
MCA: Middle cerebral artery
MIP: Maximum intensity projection
NCCT: Non-contrast computed tomography
RAPID: Rapid processing of perfusion and diffusion
TIA: Transient ischemic attack
*T*_max_: Time to maximum
